# 

**Published:** 1902-06-15

**Authors:** 


### ANNOUNCEMENTS.

### NATIONAL DENTAL ASSOCIATION.

The sixth annual session will be held in Niagara Falls, N. Y., July 28–31.

A good program is being prepared and a large and profitable meeting is anticipated.

A rate of one fare and a third for the round trip, on the certificate plan, has been secured on all roads in the United States and part of Canada.

In purchasing ticket going, full fare must be paid and a railroad *certificate* taken, this, when properly signed entitles holder to return for one-third fare.

Tickets may be bought going from July 22d to 29th and certificates for return journey may be used as late as August 4th.

---

The National Association of Dental Faculties will meet at Niagara Falls, July 24, 25 and 26, 1902.

The National Association of Dental Examiners will meet at Niagara Falls, July 25, 1902.

The Indiana Dental Association meets at Lake Maxinkuckee, June 24, 25, 26, 1902.

